# Psychometric Analysis of a German-Language Version of the Work–Family Conflict and Family–Work Conflict Scale

**DOI:** 10.3389/fpsyg.2021.782618

**Published:** 2021-12-23

**Authors:** Nikola Komlenac, Lisa Stockinger, Tanja Vogler, Margarethe Hochleitner

**Affiliations:** Gender Medicine Unit, Medical University of Innsbruck, Innsbruck, Austria

**Keywords:** Work–Family Conflict and Family–Work Conflict Scale, psychometric analysis, invariance analysis, confirmatory factor analysis (CFA), German translation, validity, reliability

## Abstract

The Work–Family Conflict and Family–Work Conflict Scale (WFC & FWC Scale) is a questionnaire commonly used to assess conflicts that arise when required time devotion and strain for work obligations interfere with family responsibilities (work-family conflict) and conflicts that arise when family responsibilities interfere with work responsiblities (family work conflict). Past reports on the psychometric properties and recommendations for application of the WFC & FWC Scale mostly rely on samples from the United States. The current study is the first to report psychometric properties of a German-language version of the WFC & FWC Scale, including invariance analyses across women and men, and test-retest reliabilities. The analysis of the latent structure that was based on responses from 274 employes (77.0% women, 23.0% men) of a medical university in Austria revealed that the bifactor model had a satisfactory fit with the data. Configural and metric invariance indicated a similar factor structure and similar meaning in women and men. However, scalar invariance cannot be assumed. Thus, differences in scale scores between women and men might not adequately reflect level differences in the underlying latent factor. High internal consistencies and high test-retest reliabilities offer evidence for adequate reliability. Additionally, evidence for convergent (links to work stress and relationship satisfaction) and divergent validity (no links to career ambition) were found. In summary, the current study offers adequate evidence for validity and reliability of a German-language version of the WFC & FWC Scale.

## Introduction

The Work–Family Conflict and Family–Work Conflict Scale (WFC & FWC Scale) (Netemeyer et al., 1996) is an instrument that is widely used to assess the so-called work-family conflict (WFC) and family work conflict (FWC) (Min et al., 2021). WFC arises when general demands of time devoted to and strain created by work interfere with family responsibilies (Greenhaus and Beutell, 1985; Netemeyer et al., 1996). FWC can be caused by family responsibilities setting time demands or causing strain that interfere with work responsiblities (Greenhaus and Beutell, 1985; Netemeyer et al., 1996). The experience of strong WFC and/or strong FWC has been associated with negative work-related outcomes (such as lower job performance, reduced job satisfaction, or feelings of exhaustion), family related outcomes (e.g., reduced leisure satisfaction, more frequent conflicts with family members), or stress/health-related outcomes (e.g., heightened anxiety, depression, and poorer general health) (Amstad et al., 2011; Sirgy and Lee, 2018; Borgmann et al., 2019; Borgmann et al., 2020).

The WFC & FWC Scale is a brief instrument that focuses mostly on assessing conflicting time demands and experienced strain caused by conflicting demands of work and family responsiblities (Netemeyer et al., 1996). Confirmatory factor analyses based on data from three different United States samples show that two-factor models had satisfactory fit indices (comparative fit index (CFI) >0.93; Tucker–Lewis Index (TLI) >0.91). Furthermore, the scales had acceptable internal consistencies (Cronbach’s alphas >0.83) (Netemeyer et al., 1996). The advantage of the WFC & FWC Scale is its short length, while still measuring two constructs, namely WFC and FWC. Furthermore, in comparison to other instruments that measure WFC (Carlson et al., 2000; Grzywacz et al., 2006), the WFC & FWC Scale has proven to have satisfactory measurement precision, especially in heterogeneous samples and when participants experience moderate levels of WFC or FWC (Min et al., 2021).

However, to authors’ knowledge psychometric analyses of a German-language version of the WFC & FWC Scale have not been reported. To close this gap in the literature, the current study reports evidence for validity and reliability of a German-language version of the WFC & FWC Scale. To this end, the WFC & FWC Scale (Netemeyer et al., 1996) was translated from English to German by a German native-speaker and professional translator. In order to test the face validity of this translation, an English native-speaker and professional translator translated the German version back to English (back-translation method) and the two English-language versions were compared (Brislin, 1970). Authors discussed any discrepancies until they reached consensus on the final wording. All the other questionnaires that were used in the current study to estimate convergent and divergent validity were developed in English (Cavanaugh et al., 2000; Funk and Rogge, 2007; Meeussen and Van Laar, 2018) and were also translated to German with the forth-and-back procedure (Brislin, 1970).

Specific indicators of reliability and validity (Döring and Bortz, 2016) that were previously estimated for the original English version of the WFC & FWC Scale have been estimated for the German-language version of the WFC & FWC Scale. First, the internal factor structure of the instrument was analyzed and internal consistencies were calculated (Netemeyer et al., 1996). Working from known associations, convergent validity was tested by calculating the correlation between the WFC & FWC Scale and experienced stress at work or relationship satisfaction. Divergent validity was tested by analyzing correlations between the WFC & FWC Scale and career ambitions (Netemeyer et al., 1996; Ellinas et al., 2018). The current study adds further to the current literature by providing test-retest reliability and by performing invariance analyses across women and men. Analyzed was whether the German-language version of the WFC & FWC Scale measures similar constructs, with similar meaning, similar levels of the latent factors, and a similar degree of precision in women and men (Kline, 2015). Knowing whether invariance can be assumed across women and men is important for the interpretation of previously reported gender differences on the WFC & FWC Scale (Shockley et al., 2017).

## Methods

### Procedures and Participants

This study was part of a larger study that was conducted from April to June 2020 (T1) among employes at an Austrian medical university (Komlenac et al., 2021). In order to estimate test-retest reliability, data were collected at a second time point from September to October 2020 (T2). Data sets were matched with the help of subject-generated identification codes (Schnell et al., 2010). All employes who followed the e-mailed invitation were asked to give informed consent before opening the online questionnaire that was hosted on *SoSci: der onlineFragebogen*^1^. The medical university’s Ethics Committee exempted the current study from full ethics review and confirmed (on April 8, 2020) that under Austrian law the current study did not require formal approval by an ethics committee [Federal Act on the Organization of Universities and their Studies (BGBl, 2002)], Hospitals and Health Resorts Act [Bundesgesetz über Krankenanstalten und Kuranstalten (KAKuG), 2016].

In total, 400 employes participated in the study at T1. Of those respondents, 274 were included for the analyses of the internal factor structure, the invariance analyses, and the convergent and divergent validity, because they passed two attention-check items (“Please select the response “Agree””) (Huang et al., 2012) and did not discontinue the survey before reaching the WFC & FWC Scale. The current study’s sample size seemed adequate, because lower bound sample size calculations revealed that a minimum sample size of *n* ≥ 156 is recommended for detecting effects of at least 0.4 with a power of 0.8 at a significance level of 0.05 in structural equation models with three latent variables and ten indicator variables (Westland, 2010). Furthermore, the current study’s sample size complies with Barrett (2007) recommendation that structural equation models should have a sample size of at least 200. Such a sample size corresponds with the median sample sizes previously used for calculation of structural equation models (MacCallum and Austin, 2000; Shah and Goldstein, 2006). The final sample included 211 women (77.0%) and 63 men (23.0%). Participants were on average 41.3 (*SD* = 10.4) years old. The majority of the participants held a university degree (71.4%), worked full time (64.2%), and in academia (54.0%). The other participants had absolved secondary school, vocational training or had a university entrance-level qualification (28.6%), worked part-time (35.8%), and in administration (46.0%). Most of the participants identified as heterosexual (93.0%), had Austrian nationality (79.6%), were in a relationship (85.4%), and had children (55.8%). The other participants did not identify as heterosexual (7.0%), were not Austrian (20.4%), were single (14.6%), and did not have children (44.2%). Responses from those 82 participants (17.1% men, 82.9% women) who filled out the WFC & FWC Scale at both time points (T1 and T2) were used to estimate the test-retest reliability.

### Measures

The Work–Family Conflict and Family–Work Conflict Scale (WFC & FWC Scale) (Netemeyer et al., 1996) consists of ten statements. Five statements describe conflicts in which the general demands of time devoted to and strain created by work interfere with family responsibilities (work-family conflict, WFC). Five statements describe conflicts in which family responsibilities interfere with work responsiblities (family work conflict, FWC) (Netemeyer et al., 1996). Participants were asked to indicate on a seven-point Likert scale whether the statements described their experiences (1 = *strongly disagree*, 7 = *strongly agree*). Higher mean scores indicated stronger WFC (Cronbach’s α = 0.88–0.89) and FWC (Cronbach’s α = 0.83–0.89) (Netemeyer et al., 1996).

Participants reported how much stress is caused (1 = *produces no stress*, 5 = *produces a great deal of stress*) by each of eleven described situations at work (Cavanaugh et al., 2000). Six items assessed challenge stressors (Cronbach’s α = 0.87), i.e., demands at work that cause stress but are associated with potential gains for the individual (e.g., number of projects and assignments) (Cavanaugh et al., 2000). Five items assessed hindrance stressors (Cronbach’s α = 0.75), i.e., demands at work that cause stress but are rarely associated with any potential gains for the individual (e.g., paper work/bureaucratic demands) (Cavanaugh et al., 2000). In the current study, challenge stressors have been assessed with an internal consistency of 0.88 and hindrance stressors with an internal consistency of 0.67.

Participants responded on a five-point Likert scale (1 = *totally disagree*, 5 = *totally agree*) to five questions asking to what extent they had career ambitions, i.e., would like to improve performance or pursue a higher position at work (Meeussen and Van Laar, 2018). Higher mean scores indicated strong career ambitions (Cronbach’s α = 0.88) (Meeussen and Van Laar, 2018). In the current study, the Career Ambition Scale had an internal consistency of 0.85.

The four-item version of the Couples Satisfaction Index (CSI-4) (Funk and Rogge, 2007) was used to assess participants’ relationship satisfaction. One item used a seven-point Likert scale (1 = *Not at all true*, 7 = *Perfect*), whereas the other items used a six-point Likert scale for responses (1 = *Extremely unhappy*, 6 = *Completely true*). Higher sum scores indicate stronger relationship satisfaction (Cronbach’s α = 0.94) (Funk and Rogge, 2007). In the current study, the internal consistency of the CSI-4 was 0.93.

Additionally, sociodemographic information was assessed with self-constructed questions about participants’ gender, age, sexual orientation, relationship status, highest level of education, nationality, position at the medical university, and number of children. For each question, participants could choose among several response options and give a free text response (Komlenac et al., 2021). For the analysis, all sociodemographic variables were dichotomized.

### Statistical Analysis

Descriptive statistics, correlations and the most widely used measure of reliability, namely Cronbach’s alpha (Deng and Chan, 2017), were calculated with SPSS, version 26.0 (IBM Corp., Armonk, NY, United States). Because McDonald’s ω (McDonald, 1999) is recommended as an alternative indicator of reliability, especially when loadings of items vary (Cho and Kim, 2015; Deng and Chan, 2017), McDonald’s ω was calculated as a measure of scale reliability. Hierarchical McDonald’s ω (ω_h_) was calculated as a measure of the general factor’s reliability (Zinbarg et al., 2005; Zinbarg et al., 2006; Revelle and Zinbarg, 2009; Chung et al., 2016). McDonald’s ωs were calculated with R (R Core Team, 2021), version 4.1.1, using the *psych* package (Revelle, 2018) and the *MBESS* package (Dunn et al., 2014; Kelley, 2018). MPlus, Version 8 (Muthén and Muthén, 1998–2017) (Muthén & Muthén, Los Angeles, CA, United States), was used to calculate structural equation models. The fit indices of the structural equation models were calculated with the robust maximum likelihood (MLR) estimator (Kline, 2015; Muthén and Muthén, 1998–2017; Yuan and Bentler, 2000), because Kolmogorov–Smirnov tests revealed that variables were not normally distributed (*D*s(274) >0.07, *p*s ≤ 0.001) (Field, 2009). The following cut-offs for the fit indices were used: *p-*value of scaled χ^2^ >0.05 (Weiber and Mühlhaus, 2014), root mean square error of approximation (RMSEA) ≤0.08 (Browne and Cudeck, 1993), standardized root mean square residual (SRMR) ≤0.1, CFI ≥0.90, and TLI ≥0.90 (Weiber and Mühlhaus, 2014).

Similar to the original and other validation studies of the WFC & FWC Scale (Netemeyer et al., 1996; Boyar et al., 2006), a one-factor model (all items loaded on a single factor) and a two-factor model (all items loaded on their respective factor) were calculated. Furthermore, in order to test whether items measure two constructs that can be assumed to represent one broad central construct (i.e., a “general factor”) a bifactor model (all items loaded on a general factor in addition to their respective factor) was tested (Chen et al., 2012; Reise, 2012; Rodriguez et al., 2016). For the model with best fit indices, configural, metric, scalar, and residual invariance were tested (Kline, 2015). Models were compared with scale-adjusted chi-square difference tests (Satorra and Bentler, 2001; Satorra and Bentler, 2010)^2^ and changes in CFI (ΔCFI). Non-significant chi-square difference tests and ΔCFI ≤0.01 indicate equivalent models (Cheung and Rensvold, 2002). Additionally, bias-corrected bootstrap confidence intervals (bootstrap sample *n* = 1,000) were calculated in order to analyze similarities of factor loadings and intercepts between women’s and men’s structural equation models (Cheung and Lau, 2012).

## Results

### Descriptive Statistics

On average, participants did *rather not* experience WFC and most reported not experiencing FWC (Table 1), whereby men experienced both kinds of conflict more often than did women (Table 1). Cronbach’s alphas and McDonald’s ωs indicated satisfactory internal consistencies (Ponterotto and Ruckdeschel, 2007; Table 1). The hierarchical McDonald’s ω (ω_h_; McDonald, 1999) was 0.70 in the total sample, 0.65 in the male subsample, and 0.67 in the female subsample. Test-retest reliabilities were above 0.70 (Table 1). In women, the WFC & FWC Scale correlated with experienced work stress, but were unrelated to career ambitions or relationship satisfaction (Table 2). In men, the WFC & FWC Scale also correlated with experienced work stress, but not with career ambitions. Larger FWC went along with reduced relationship satisfaction in men (Table 2).

**TABLE 1:** Means, standard deviations, Cronbach’s alphas, and McDonald’s omegas by gender.

Scales	T1		T2		*r*_test–retest_	α	ω (95%*CI*)	Men			Women			*t*(272)	*d*
	*M*	*SD*	*M*	*SD*				*M*	*SD*	ω (95%*CI*)	*M*	*SD*	ω (95%*CI*)		
WFC	3.4	1.6	3.3	1.7	0.73**	0.92	0.92 (0.90–0.94)	4.1	1.4	0.91 (0.84–0.95)	3.2	1.6	0.92 (0.90–0.94)	4.0**	0.6
FWC	2.4	1.3	2.3	1.4	0.79**	0.90	0.90 (0.87–0.93)	2.8	1.4	0.93 (0.89–0.96)	2.2	1.3	0.89 (0.85–0.92)	3.1*	0.4
WC	2.9	1.3	2.8	1.4	0.81**	0.92	0.92 (0.90–0.93)	3.5	1.2	0.90 (0.83–0.94)	2.7	1.6	0.91 (0.89–0.93)	4.1**	0.6

Scale scores ranged from 1 to 7. Higher scores indicate greater work-family conflict or family work conflict. α, Cronbach’s alpha coefficient; ω, McDonald’s ω; WFC, work-family conflict; FWC, family work conflict; WC, work-related conflict; T1, Time point 1; T2, Time point 2; ω, McDonald’s ω. *p < 0.01, **p < 0.001.

**TABLE 2:** Convergent and discriminant validity.

Scales	(1)	(2)	(3)	(4)	(5)	(6)	(7)
(1) WFC				0.59***	0.43***	–0.11	0.08
(2) FWC				0.40**	0.46***	−0.34*	0.15
(3) WC				0.57***	0.53***	−0.27*	0.13
(4) Stress hindrance	0.61***	0.42***	0.60***				
(5) Stress challenge	0.46***	0.34***	0.46***				
(6) CSI	0.09	–0.10	0.00				
(7) Career ambition	0.03	–0.03	0.00				

Below diagonal correlations in women (n = 211) are reported; above diagonal correlations in men (n = 63) are reported. WFC, work-family conflict; FWC, family work conflict; WC, work-related conflict; CSI, Couples Satisfaction Index. *p < 0.050, **p < 0.010, ***p < 0.001.

### Confirmatory Factor Analysis

The one-factor model did not satisfactorily fit the data (Table 3). The two-factor model and the bifactor model had satisfactory fit indices, whereas the bifactor model proved to have better fit indices (Table 3). In the bifactor model, all items significantly loaded to their respective factor (λ = 0.43 –0.67) and to the general factor (λ = 0.50 –0.76).

**TABLE 3:** Goodness-of-fit indices of the models (*N* = 274).

Model	Fit indices						
	S-B χ^2^	df	Scaling correction	CFI	TLI	RMSEA [90% CI]	SRMR
(1) 1-Factor	374.7**	35	1.558	0.712	0.630	0.188 [0.171–0.206]	0.128
(2) 2-Factor	101.7**	36	1.340	0.944	0.930	0.082 [0.063–0.101]	0.146
(3) Bifactor	47.6*	28	1.327	0.983	0.973	0.051 [0.024–0.075]	0.058

**Model comparison**	**Δχ^2^**	**df**	***p***	**ΔCFI**	**Conclusion**		

1 vs. 2	71.1	1	<0.001	0.232	Prefer 2		
2 vs. 3	52.8	8	<0.001	0.039	Prefer 3		

The conclusion in the model comparison section is based on a joint consideration of Δχ^2^ and ΔCFI. Chi-square statistics were estimated with the robust maximum likelihood (MLR) estimator and Δχ^2^ was calculated with the scale-adjusted chi-square difference test (http://www.thestatisticalmind.com/calculators/SBChiSquareDifferenceTest.htm, accessed 2021-09-06). df, degrees of freedom; RMSEA, root mean square error of approximation; SRMR, standardized root mean square residual; TLI, Tucker–Lewis Index; CFI, comparative fit index; CI, confidence interval. *p < 0.05, **p < 0.001.

### Invariance Analysis

The bifactor model had acceptable fit indices in women and men. Additionally, the model of configural invariance had acceptable fit indices (Table 4). Metric invariance was supported because the model for metric invariance did not have poorer fit indices than did the configural model. Additionally, factor loadings in women and men were similar (d_ifferece_ = −0.2 – 0.3; 95%[lower limit (LL): −1.4 – −0.3; upper limit (UL) 0.3 – 1.6]) (Cheung and Lau, 2012). However, scalar invariance was not supported because the scalar model had significantly poorer fit indices than did the metric model (Table 4). This conclusion was supported by the finding that all intercepts (except one) were significantly different in women and men (d_ifferece_ = −0.3 – 1.1; 95%[−0.2 – 0.6; 0.8 – 1.5]) (Cheung and Lau, 2012). Because scalar invariance was therefore not assumed, residual invariance was not tested (Kline, 2015).

**TABLE 4:** Measurement invariance across women and men.

Model	Fit indices						
	S-B χ^2^	df	Scaling correction	CFI	TLI	RMSEA [90% CI]	SRMR
Bifactor men	27.4	28	0.988	1.000	1.003	0.000 [0.00 –0.095]	0.044
Bifactor women	49.6*	28	1.261	0.974	0.959	0.046 [0.061–0.088]	0.050
(1) Configural	79.7*	56	1.124	0.981	0.970	0.056 [0.023–0.082]	0.049
(2) Metric	86.7	73	1.190	0.989	0.986	0.037 [0.000–0.064]	0.067
(3) Scalar	115.2*	83	1.170	0.974	0.972	0.053 [0.026–0.075]	0.102

**Model comparison**	**Δχ^2^**	**df**	***P***	**ΔCFI**	**Conclusion**		

1 vs. 2	9.7	17	0.917	0.008	Equivalent		
2 vs. 3	30.9	10	<0.001	0.015	Not equivalent		

The conclusion in the model comparison section is based on a joint consideration of Δχ^2^ and ΔCFI. Chi-square statistics were estimated with the robust maximum likelihood (MLR) estimator and Δχ^2^ was calculated with the scale-adjusted chi-square difference test (http://www.thestatisticalmind.com/calculators/SBChiSquareDifferenceTest.htm, accessed 2021-09-06). df, degrees of freedom; RMSEA, root mean square error of approximation; SRMR, standardized root mean square residual; TLI, Tucker–Lewis Index; CFI, comparative fit index; CI, confidence interval. *p < 0.05.

## Discussion

The current study is the first to report psychometric properties of a German-language version of the widely used Work–Family Conflict and Family–Work Conflict Scale (WFC & FWC Scale) (Netemeyer et al., 1996). Similar to the original English version of the WFC & FWC Scale, the German-language version of the scale is characterized by having high internal consistencies in women and men (Netemeyer et al., 1996; Ponterotto and Ruckdeschel, 2007). An indicator of construct validity of the German-language version of the WFC & FWC Scale is the good fit of data to the two-factor model, comparable to results of validation studies of the original version of the WFC & FWC Scale (Netemeyer et al., 1996). Additionally, the current study revealed that the bifactor model has an even better fit to the data, indicating that the WFC & FWC Scale measures “distinct but related forms of interrole conflict” (Netemeyer et al., 1996). The good fit of the bifactor model and the relatively high hierarchical McDonald’s ω also support the calculation of a general score for the WFC & FWC Scale (Reise et al., 2013). Such a general score proved to have satisfactory internal consistency and test-retest reliability (Table 1) (Ponterotto and Ruckdeschel, 2007).

However, arguments against using such a general score offer past research that found that WFC and FWC can have different consequences (Amstad et al., 2011). Whereas WFC was found to be mostly linked to negative work-related outcomes, FWC is more likely associated with family related outcomes (Amstad et al., 2011). In line with this past research, the current study showed FWC but not WFC to be linked to relationship satisfaction in men. With those findings and found associtions between the WFC & FWC Scale and work stress, the current study shows evidence for convergent validity (Netemeyer et al., 1996). Divergent validity was supported by the fact that no links were found between the WFC & FWC Scale and career ambitions (Ellinas et al., 2018).

The current study’s invariance analysis revealed, similar to a meta-analysis of the WFC & FWC Scale, that configural and metric invariance can be assumed across women and men (Shockley et al., 2017). Those results support the conclusion that the two scales making up the WFC & FWC Scale have similar meaning across women and men (Gregorich, 2006). However, current and previous findings (Shockley et al., 2017) suggest the presence of a differential additive (acquiescence) response style (Cheung and Rensvold, 2000; Kline, 2015). The finding that scalar invariance cannot be assumed and that all intercepts (except one) were significantly different in women and men (Cheung and Lau, 2012) indicates that one group is systematically giving higher or lower responses than the other group. Hence, a given score may not represent the same level of a latent factor in women and men. This affects the general score and the two scale scores (WFC and FWC). Thus, the currently found gender differences and past reported gender differences (e.g., Shockley et al., 2017) need to be interpreted with caution, because differences in mean scores may not represent differences on the (underlying) latent level of experienced WFC or FWC.

The current study is one of the first studies to report test-retest reliabilities of the WFC & FWC Scale (Min et al., 2021). Obtained estimates of the test-retest reliabilities for the German version of the WFC & FWC Scale can be interpreted as being acceptable (Nunnally and Bernstein, 1994; Vilagut, 2014) and exceed test-retest reliabilities reported for similar instruments assessing WFC or FWC (Fisher et al., 2016; Min et al., 2021). Thus, the German-language version of the WFC & FWC Scale can be recommended for future longitudinal studies that analyze (systematic) changes in WFC or FWC over time (Borgmann et al., 2020).

The generalizability of the current study’s findings is limited because the study’s sample consisted of employes of only one organization. Even though lower bound sample size calculations and recommendations concerning minimum sample sizes justify the adequacy of the current study’s sample size, this relatively small sample size further limits the generalizability of the findings (Barrett, 2007; Westland, 2010). Small sample sizes do not only reduce the trustworthiness of results, but also increase the risk for technical problems during the analysis (Kline, 2015). Thus, future studies of the WFC & FWC Scale should include larger samples. Future studies employing larger and more diverse samples are needed to test measurement invariance across groups additional to women and men, such as groups with different cultural background, age, sexual orientation, or gender identity (including participants who identify with a gender identity other than *cis*-women and *cis*-men). In the current study not only the WFC & FWC Scale was translated from English to German, but also the other instruments that were used to estimate convergent and divergent validity were available only in English and needed to be translated for the current study. Those other instruments have not been subject to the same rigorous analyses of psychometric properties as was the WFC & FWC Scale. Thus, results concerning the convergent and divergent validity need to be interpreted with caution until future studies report on the psychometric properties of all the currently used instruments. Another limitation of the study is the absolutist approach, which is based on the assumption that a translated measure will be identical to the original version in all aspects (Swami and Barron, 2019). However, during translation more rigorous and more culture-specific adaptations might be needed for the translated version to be conceptually equivalent in a cultural context for which the original version was not developed (Swami and Barron, 2019). After the translation of items pre-testing in a relatively small sample can be conducted to assess with quantitative and qualitative methods the understanding, acceptability, and emotional impact of the culturally adapted items (Swami and Barron, 2019). Finally, as is the case with most questionnaire studies, the current study is based on self-reports and is therefore susceptible to socially desirable responding.

Despite its limitations the current study offers adequate evidence for validity and reliability of a German-language version of the Work–Family Conflict and Family–Work Conflict Scale for the assessment of two distinct but related constructs, namely WFC and FWC (Netemeyer et al., 1996). The found psychometric properties of the WFC & FWC Scale also support the calculation of a general score for future studies that do not need to distinguish between WFC and FWC. Mean differences between women and men should be interpreted with caution because scalar invariance cannot be assumed. Future studies might improve the scale further by analyzing cultural appropriateness and developing items that are especially relevant and normative for specific target populations (Swami and Barron, 2019).

## Data Availability Statement

The raw data supporting the conclusions of this article will be made available by the authors, without undue reservation.

## Ethics Statement

Ethical review and approval was not required for the study on human participants in accordance with the local legislation and institutional requirements. The patients/participants provided their written informed consent to participate in this study.

## Author Contributions

NK, LS, and MH designed the research. NK and MH collected the data. NK analyzed and interpreted the data and wrote the manuscript. TV critically commented the manuscript. All authors read and approved the final manuscript.

## Conflict of Interest

The authors declare that the research was conducted in the absence of any commercial or financial relationships that could be construed as a potential conflict of interest.

## Publisher’s Note

All claims expressed in this article are solely those of the authors and do not necessarily represent those of their affiliated organizations, or those of the publisher, the editors and the reviewers. Any product that may be evaluated in this article, or claim that may be made by its manufacturer, is not guaranteed or endorsed by the publisher.

## Footnotes

1
http://soscisurvey.de/

2 http://www.thestatisticalmind.com/calculators/SBChiSquareDifferenceTest.htm (accessed 2021-09-06)
